# Detection of mercury ions using silver telluride nanoparticles as a substrate and recognition element through surface-enhanced Raman scattering

**DOI:** 10.3389/fchem.2013.00020

**Published:** 2013-10-09

**Authors:** Chia-Wei Wang, Zong-Hong Lin, Prathik Roy, Huan-Tsung Chang

**Affiliations:** Department of Chemistry, National Taiwan UniversityTaipei, Taiwan

**Keywords:** surface-enhanced Raman scattering, nanoparticles, silver telluride, mercury telluride, mercury ions

## Abstract

In this paper we unveil a new sensing strategy for sensitive and selective detection of Hg^2+^ through surface-enhanced Raman scattering (SERS) using Ag_2_Te nanoparticles (NPs) as a substrate and recognition element and rhodamine 6G (R6G) as a reporter. Ag_2_Te NPs prepared from tellurium dioxide and silver nitrate and hydrazine in aqueous solution containing sodium dodecyl sulfate at 90°C with an average size of 26.8 ± 4.1 nm (100 counts) have strong SERS activity. The Ag_2_Te substrate provides strong SERS signals of R6G with an enhancement factor of 3.6 × 10^5^ at 1360 cm^−1^, which is comparable to Ag NPs. After interaction of Ag_2_Te NPs with Hg^2+^, some HgTe NPs are formed, leading to decreases in the SERS signal of R6G, mainly because HgTe NPs relative to Ag_2_Te NPs have weaker SERS activity. Under optimum conditions, this SERS approach using Ag_2_Te as substrates is selective for the detection of Hg^2+^, with a limit of detection of 3 nM and linearity over 10–150 nM. The practicality of this approach has been validated for the determination of the concentrations of spiked Hg^2+^ in a pond water sample.

## Introduction

Monitoring the level of mercury ions in ecological systems is an extremely important issue, mainly because they are highly toxic, non-biodegradable, and bioaccumulated (Zahir et al., 2005; Clarkson et al., 2008). Several techniques such as atomic absorption/emission spectrometry, atomic fluorescence spectrometry, inductively coupled plasma spectrometry (ICP-MS) have been applied to detect Hg^2+^ in environmental and biological samples (Leermakers et al., 2005; Butler et al., 2006; Li et al., 2006). Among them, ICP-MS provides the highest sensitivity and a widest linear range, but the system is expensive because it requires expensive noble gas. In addition, it is not easy for the in-field analysis.

With advantages of simplicity, low cost, sensitivity, and selectivity, a number of optical and electrochemical sensors have been demonstrated for the detection of Hg^2+^ (Huang and Chang, 2007; Darbha et al., 2008; Jena and Raj, 2008; Zhu et al., 2009). Having extremely high stability, specificity, and ease in preparation, DNA-based optical sensors have become popular for the detection of Hg^2+^ (Chiang et al., 2008; Stewart et al., 2008; Dave et al., 2010). Polythymines (*T_n_*) that are specific toward Hg^2+^ ions through T–Hg^2+^–T coordination have been used for the selective and sensitive detection of Hg^2+^ ions through fluorescence detection based on the analyte induced changes in the DNA conformation, leading to enhanced efficiency in the fluorescence resonance energy transfer between the donor and the acceptor or increased quantum yield of the fluorophore. In addition, *T_n_* conjugated with gold nanoparticles (NPs) have been used for the detection of Hg^2+^ ions through absorption or fluorescence modes (Huang et al., 2007; Lee et al., 2007; Wang et al., 2008; Xue et al., 2008). The detections are mainly based on the analyte induced changes in the DNA conformation and charge density on the surfaces of the Au NPs, leading to changes in absorbance (red shift upon aggregation) or fluorescence intensity (either decreases or increases). Surface-enhanced Raman scattering (SERS) using Ag NPs conjugated with *T_n_* and organic dyes (reporter) are alternative for the sensitive detection of Hg^2+^ (Wang et al., 2009, 2011). Upon interaction with Hg^2+^, the DNA conformation changes, leading to changes in the distance of the reporter from the surfaces of Ag NPs and thus changes in the SERS signal (Grubisha et al., 2003; Doering et al., 2007). Although these sensing systems are sensitive and suitable for in-field analysis, the DNA is expensive and their sensitivity and selectivity are highly dependent on the ionic strength of the samples, limiting their wide practicality.

Relative to normal Raman scattering, SERS can provide enhancement factors (EFs) up to 10^15^ theoretically through a long-range electromagnetic (EM) effect such as “hot spots,” and/or chemical effect due to the charge-transfer excitation of chemisorbed molecules (Aravind et al., 1981; Kneipp et al., 1997; Nie and Emery, 1997). The EF values are dependent on the compositions, sizes, and shapes of the SERS substrates. Relative to spherical shaped silver NPs, silver plates and silver nanowires provides higher EF values; EF values up to 10^8^ have been demonstrated for the SERS signals of common reporters such as Rhodamine 6G (R6G), 4-mercaptobenzoic acid, and 5,5′-dithiobis(2-nitrobenzoic acid) (Tao et al., 2003; Yang et al., 2007). Gold-tellurium nanodumbbells, gold-tellurium nanopeapods, and gold pearl-necklace nanomaterials (Au PNNs) providing EF values of R6G up to 5.6 × 10^9^ have been used for the selective detection of human serum albumin down to 70 pM using AB 580 as a reporter (Lin and Chang, 2008; Lin et al., 2011).

In this study, we developed a simple SERS approach using silver telluride (Ag_2_Te) NPs as substrates for sensitive and selective detection of Hg^2+^ in aqueous solutions. Ag_2_Te NPs were prepared from tellurium dioxide and silver nitrate in the presence of hydrazine and sodium dodecyl sulfate (Samal and Pradeep, 2009). The Ag_2_Te NPs provided SERS enhancement effect of R6G. Upon increasing the concentration of Hg^2+^ ions, the SERS signal of R6G decreased. The novel SERS approach was further validated by the determination of the concentrations of Hg^2+^ in pond water samples, showing advantages of sensitivity, selectivity, and simplicity.

## Materials and methods

### Chemicals

Hydrazine monohydrate (80%) and tellurium dioxide powder (99.9%) were purchased from SHOWA (Tokyo, Japan). Sodium phosphate monobasic, dibasic, and tribasic, and sodium dodecyl sulfate were purchased from Acros (Geel, Belgium). Mercury chloride, R6G, silver nitrate, and other metal salts [Ca^2+^, Co^2+^, Cu^2+^, Cd^2+^, K^+^, Mg^2+^, Mn^2+^, Ni^2+^, Cr^3+^, Fe^3+^, Fe^2+,^ Pd^2+^, Zn^2+^, Pb^2+^, and Na^+^ (chlorides)] used in this study were purchased from Sigma Aldrich (Missouri, USA). Ultrapure water was obtained using a Milli-Q ultrapure (18.2 MΩ-cm) system.

### Synthesis of Ag_2_Te NPs

Hydrazine (1 mL) was added slowly to a sample vial containing aqueous solution (9 mL) of tellurium dioxide (5 mM), silver nitrate (10 mM), and sodium dodecyl sulfate (30 mM). The mixture was then subjected to constant magnetic stirring at 90°C. The solution changed color from colorless to dark brown after 8 h, indicating the formation of Ag_2_Te NPs. To terminate the reaction and to remove most of the matrix (e.g., hydrazine), the Ag_2_Te NPs were subjected to three cycles of centrifugation [relative centrifugation force (RCF): 12000 g for 10 min] and wash (3 × 10 mL of water). For simplicity, the concentration of the as-prepared Ag_2_Te NPs in 10 mL H_2_O is represented as 1 X.

### Characterization

JEOL JSM-1230 and FEI Tecnai-G2-F20 transmission electron microscopes (TEM) were used to measure the sizes and shapes of the as-prepared Ag_2_Te NPs. The re-dispersed Ag_2_Te NPs were separately placed on formvar/carbon film Cu grids (200 mesh; Agar Scientific) and dried at ambient temperature (25°C). An energy dispersive X-ray (EDAX) system (Inca Energy 200, Oxford) was used to determine the composition of the as-prepared NMs. Raman spectra were recorded using a Raman spectrometer (DongWoo 500i, Korea) equipped with a 50× objective and a charge-coupled detector. The excitation wavelength was 532 nm and the spectral aperture was 50 μm. The signal collection time for each sample was 30 s.

### Detection of Hg^2+^ ions using Ag_2_Te NPs

Ag_2_Te NPs (0.01 ×, 100 μL), phosphate buffer (PB) (1 mM, pH 4.0, 100 μ L), and SDS (0.1 mM, 100 μ L) were added to aqueous solutions (0.7 mL) containing various concentrations of HgCl_2_ (final concentrations 10–150 nM). The mixtures were equilibrated under constant stirring at 37°C for 10 min. After centrifugation at 12000 g for 10 min, the supernatants were discarded and the pellets were dispersed in R6G solution (10 μ M, 20 μ L). Finally, drops (1 μ L) of the R6G mixtures were added onto separate silica wafers and dried at ambient temperature (25°C) prior to SERS measurement.

### Analysis of real sample

Pond water sample was collected from the National Taiwan University campus, and subsequently filtered through a 0.45 μm membrane. For comparison, aliquots (0.1 mL) of the pond water sample was mixed with HNO_3_ (0.9 mL, final concentration 2%) prior to ICP-MS analysis. Aliquots of the pond water (100 μ L) were spiked with standard solutions (100 μ L) containing Hg^2+^ at various concentrations (0.3–1.5 μM). Next, PB (1 mM, pH 4.0, 100 μ L), SDS (0.1 mM, 100 μ L), Ag_2_Te NPs (0.01 ×, 100 μ L), and water (500 μ L) were added to the mixture to give final volumes of 1 mL. The mixtures were equilibrated under constant stirring at 37°C for 10 min. After centrifugation at 12000 g for 10 min, the supernatants were discarded and the pellets were dispersed in R6G solution (10 μ M, 20 μ L). Finally, drops of solutions (1 μ L) were added onto separate silica wafers and dried at ambient temperature prior to SERS measurement.

## Results and discussion

### Sensing strategy

**Scheme 1:**
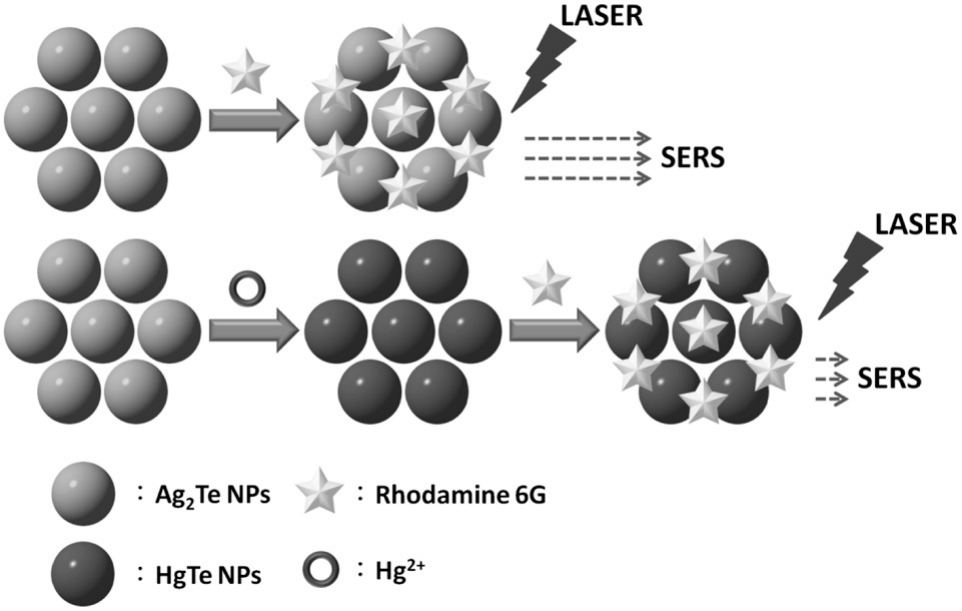
**Schematic representation of the sensing of Hg^2+^ through SERS using Ag_2_Te NPs as a substrate and recognition element**.

Scheme 1 shows the detection of Hg^2+^ based on differential SERS EFs of Ag_2_Te and HgTe NPs. Ag_2_Te relative to HgTe provides a higher EF value. The displacement reaction between Hg^2+^ and Ag_2_Te NPs leads to the formation of HgTe nanostructures and decomposition of Ag_2_Te NPs. As a result, the SERS signal of R6G decreases upon increasing the concentration of Hg^2+^, mainly because Ag_2_Te relative to HgTe NPs provides a greater SERS EF value. Although the *K*_sp_ values of Ag_2_Te and HgTe are unavailable, the latter has a small one based on that of the metal selenides (Moon et al., 2010). For example, Ag_2_Se relative to HgSe has a higher *K*_sp_ value (1 × 10^−54^ vs. 4 × 10^−59^) (Wang et al., 2007). It has been reported that the reaction of Ag_2_Te nanostructures with Hg^2+^ions is spontaneous and fast (Samal and Pradeep, 2011).

**Figure 1:**
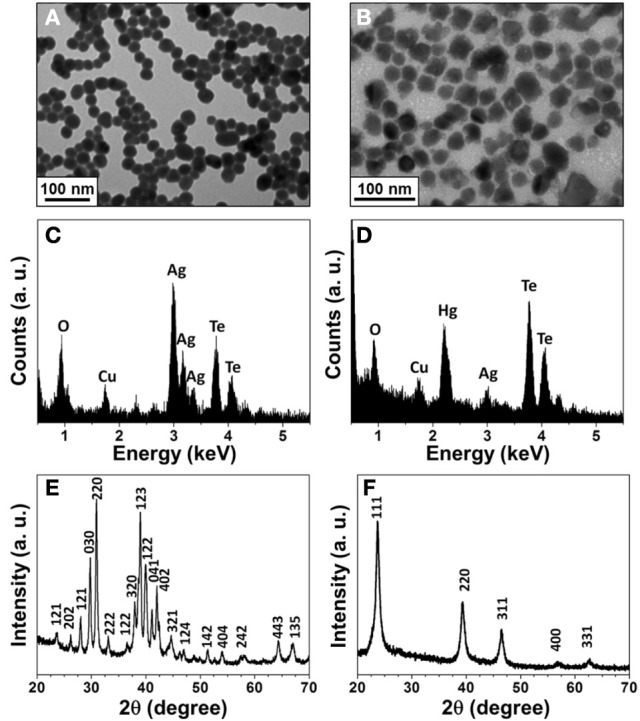
**Characterization of the synthesized Ag_2_Te and HgTe NPs. (A)** and **(B)** TEM images, **(C)** and **(D)** EDAX spectra, **(E)** and **(F)** XRD spectra of Ag_2_Te and HgTe NPs, respectively. The concentrations of Ag_2_Te and Hg^2+^ ions are 1 × and 5 mM, respectively.

To confirm the formation of HgTe NPs from the reaction of Ag_2_Te NPs with Hg^2+^, we conducted TEM, EDX, and XRD measurements. Figure 1A shows the TEM image of as-prepared Ag_2_Te NPs with an average size of 26.8 ± 4.1 nm (100 counts). On the other hand, the TEM image displayed in Figure 1B shows HgTe NPs with an average size of 37.7 ± 6.8 nm. Different morphologies in the two TEM images reveal the changes in the Ag_2_Te NPs. The EDX patterns (Figures 1C,D) confirm the displacement reaction between Hg^2+^ and Ag_2_Te NPs. The XRD patterns of Ag_2_Te NPs (Figure 1E) and its reaction product (Figure 1F) with Hg^2+^ agree with the literature data of Ag_2_Te (JCPDS: 34-0142) and HgTe (JCPDS: 75-2084), respectively.

The EF value of Ag_2_Te NPs (0.1 X) was investigated using R6G as a reporter. The limits of detection (LODs) at a signal-to-noise (S/N) 3 for R6G using silica wafers and Ag_2_Te NPs as SERS substrates were 1.8 mM and 5 nM, respectively, which reveals that the Ag_2_Te substrate provided an EF value of 3.6 × 10^5^. The EF value of Ag_2_Te NPs is comparable with that of Ag NPs (Wang et al., 1980; Kerker, 1987). The SERS signal of R6G on the Ag_2_Te substrate was about 5-fold greater than that on an HgTe substrate that had been formed from the reaction of Ag_2_Te NPs with 100 nM Hg^2+^ ions. The decreased SERS signal of R6G at a constant concentration (10 μ M) is related to the concentration of Hg^2+^ ions (to be discussed later), revealing the potential use of this approach for the determination of the concentration of Hg^2+^ ions. The decrease in SERS signal was due to the decreased amount of Ag_2_Te NPs.

### Optimization of detection conditions

**Figure 2:**
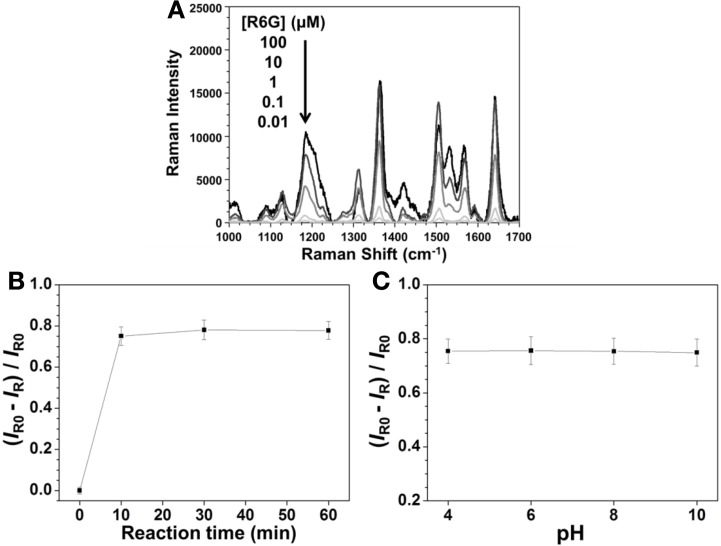
**Effects of (A) R6G concentration, (B) reaction times, and (C) pH on the SERS signal of R6G at 1361 cm^−1^ when using Ag_2_Te NPs as a substrate.** The concentrations of Ag_2_Te NPs were both 0.001 × in **(A)** and **(B)**, of Hg^2+^ were 0 in **(A)** and 100 nM in **(B)** and **(C)**, and of R6G were both 10 μM in **(B)** and **(C)**. The pH values were both 4.0 in **(A)** and **(B)** and reaction times were both 10 min in **(A)** and **(C)**. *I_R0_* and *I_R_* are the SERS intensities of R6G at 1361 cm^−1^ in the absence and presence of Hg^2+^ (100 nM), respectively, in **(B)** and **(C)**.

In order to optimize the sensing condition, we investigated several important parameters, including concentration of R6G, reaction time, and pH. The strongest SERS signal of R6G at 1361 cm^−1^ (aromatic C-C stretching) was used to evaluate the effects of these parameters. Figure 2A shows that the SERS signal of R6G at 1361 cm^−1^ increased upon increasing its concentration, with a saturated concentration of 10 μ M. Figure 2B displays the reaction was completed within 10 min when using Hg^2+^ at the concentration of 100 nM. Figure 2C displays that pH is not an important factor over the investigated pH range (4.0–10.0), mainly because the replacement reaction between Ag_2_Te and Hg^2+^ ions and the SERS signal of R6G are both not pH sensitive. Although larger Ag_2_Te NPs provided greater SERS EF values, poor reproducibility of SERS signals due to their instability in aqueous solution is problematic.

### Sensitivity and selectivity of Hg^2+^ detection

**Figure 3:**
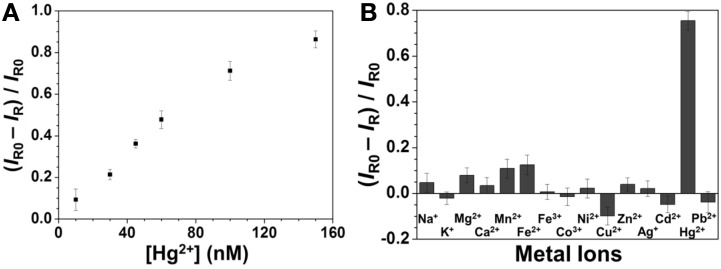
**(A)** Sensitivity and **(B)** selectivity of the SERS approach for Hg^2+^. Concentrations: 100 nM for Hg^2+^ and 1 μM for the other metal ions. Reaction time was 10 min and pH value was 4.0. The other conditions are the same as in Figure 2.

Figure 3A shows that the SERS signal of R6G decreased upon increasing the Hg^2+^ concentration, with a linear relationship between the SERS ratios ((*I_R0_*—*I_R_*)/*I_R0_*) at 1361 cm^−1^ and the Hg^2+^ concentration ranging from 10 to 150 nM (*R*^2^ = 0.98). This approach provided an LOD at an S/N = 3 of 3 nM for Hg^2+^ ions. The sensitivity is better than that provided by SERS approaches using different reporters (Zamarion et al., 2008; Han et al., 2010; Senapati et al., 2011; Luo et al., 2012). Control experiments were carried out to test the specificity of the developed approach for Hg^2+^ ions (100 nM) under optimal conditions in the presence of various metal ions (each at a concentration of 1 μM). The results displayed in Figure 3B reveal that the sensing approach is selective to Hg^2+^ ions. The potential interferences could not replace Ag^+^ ions from the Ag_2_Te NPs, resulting in negligible changes in the SERS signal of R6G at 1361 cm^−1^.

**Figure 4:**
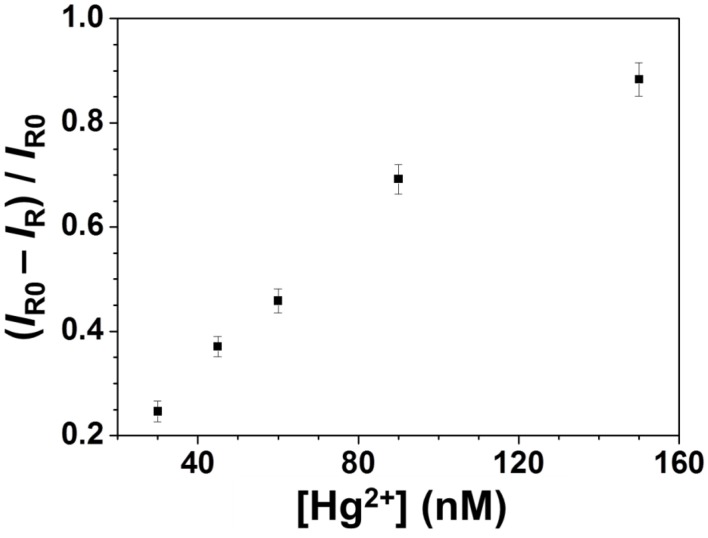
**Detection of Hg^2+^ in a spiked pond water sample through SERS using Ag_2_Te NPs as a substrate.** Concentrations of Hg^2+^ ions ranged from 30 to 150 nM.

### Real sample analysis

To examine the practicality of our approach, the concentration of Hg^2+^ in a pond water sample was determined. Our ICP-MS result showed that no Hg^2+^ was detected. By applying a standard addition method, the Raman ratios against the concentration of Hg^2+^ was found to be linear (*R*^2^ = 0.98) over 30–150 nM (Figure 4). The recovery percentage values of Hg^2+^ over the concentration range (30–150 nM) were 96–103%, showing low matrix interference. Our results reveal that this approach holds great potential for the determination of the concentrations of Hg^2+^ in environmental samples.

## Conclusions

We have demonstrated a SERS-based approach for the detection of Hg^2+^ ions using Ag_2_Te NPs as a substrate and recognition element and R6G as a reporter. To the best of our knowledge, this is the first example using a single material (Ag_2_Te) as the substrate and recognition element in SERS technology. Relative to Ag_2_Te, HgTe is a less SERS active substrate, thus the SERS signals of R6G decreased upon increasing Hg^2+^ concentration when using Ag_2_Te NPs as a substrate. This novel approach is sensitive (LOD 3 nM) and selective for the detection of Hg^2+^ ions over a wide pH range. With its high sensitivity, selectivity, and simplicity, the SERS-based approach holds great potential for the determination of the concentrations of Hg^2+^ in environmental samples.

## Author contributions

Chia-Wei Wang: He did the characterization of Ag_2_Te nanoparticles and the detection of Hg^2+^. Zong-Hong Lin: He developed the method to synthesize Ag_2_Te nanoparticles and designed the sensing strategy. Prathik Roy: He gave some advice about synthesis of Ag_2_Te nanoparticles and sensing strategy of Hg^2+^. Huan-Tsung Chang: He is the advisor of this group.

### Conflict of interest statement

The authors declare that the research was conducted in the absence of any commercial or financial relationships that could be construed as a potential conflict of interest.
